# Machine Learning Model Validated to Predict Outcomes of Liver Transplantation Recipients with Hepatitis C: The Romanian National Transplant Agency Cohort Experience

**DOI:** 10.3390/s23042149

**Published:** 2023-02-14

**Authors:** Mihai Lucian Zabara, Irinel Popescu, Alexandru Burlacu, Oana Geman, Radu Adrian Crisan Dabija, Iolanda Valentina Popa, Cristian Lupascu

**Affiliations:** 1 Faculty of Medicine, University of Medicine and Pharmacy “Grigore T Popa”, 700115 Iasi, Romania; 2 Department of Surgery, St. Spiridon Emergency Hospital, 700111 Iasi, Romania; 3 Fundeni Clinical Institute, 022328 Bucharest, Romania; 4 Center for Excellence in Translational Medicine, 022328 Bucharest, Romania; 5 Institute of Cardiovascular Diseases, 700503 Iasi, Romania; 6 The Computer, Electronics and Automation Department, Faculty of Electrical Engineering and Computer Science, University Stefan cel Mare, 720229 Suceava, Romania; 7 Pulmonology Department, Clinic of Pulmonary Diseases, 700115 Iasi, Romania

**Keywords:** liver transplantation, transplant recipients, hepatitis C, machine learning, prediction model

## Abstract

Background and Objectives: In the early period after liver transplantation, patients are exposed to a high rate of complications and several scores are currently available to predict adverse postoperative outcomes. However, an ideal, universally accepted and validated score to predict adverse events in liver transplant recipients with hepatitis C is lacking. Therefore, we aimed to establish and validate a machine learning (ML) model to predict short-term outcomes of hepatitis C patients who underwent liver transplantation. Materials and Methods: We conducted a retrospective observational two-center cohort study involving hepatitis C patients who underwent liver transplantation. Based on clinical and laboratory parameters, the dataset was used to train a deep-learning model for predicting short-term postoperative complications (within one month following liver transplantation). Adverse events prediction in the postoperative setting was the primary study outcome. Results: A total of 90 liver transplant recipients with hepatitis C were enrolled in the present study, 80 patients in the training cohort and ten in the validation cohort, respectively. The age range of the participants was 12–68 years, 51 (56,7%) were male, and 39 (43.3%) were female. Throughout the 85 training epochs, the model achieved a very good performance, with the accuracy ranging between 99.76% and 100%. After testing the model on the validation set, the deep-learning classifier confirmed the performance in predicting postoperative complications, achieving an accuracy of 100% on unseen data. Conclusions: We successfully developed a ML model to predict postoperative complications following liver transplantation in hepatitis C patients. The model demonstrated an excellent performance for accurate adverse event prediction. Consequently, the present study constitutes the foundation for careful and non-invasive identification of high-risk patients who might benefit from a more intensive postoperative monitoring strategy.

## 1. Introduction

Recent medical history has proved that, in an extended sense, humans have “spare parts” [1]. There is undoubtedly a mythical and ethical emotion-triggered philosophy regarding human organ transplantation and the “meaning of life” concept. Although it was impossible to even imagine during the heuristic era of medicine, nowadays organ transplant is no longer “magic” or an “in extremis” procedure but a logical and lifesaving, almost “common” treatment [2].

The preoccupation for finding a means of using the organs of a human being to save another human being is, of course, not new; in fact, it is as old as the first surgical attempts performed by Egyptian or Indian doctors with skin grafts. Nevertheless, it was only in 1959 that Louisiana’s Charity Hospital performed the first successful kidney transplant between twins [3], followed by the first liver transplant in 1967 at the University of Colorado, performed by Dr. Thomas Starzl, with an unfortunate brief post-procedure survival of the patient [4]. After discovering cyclosporine, the post-transplant survival rate improved significantly [3].

Today, a liver transplant is a leading indication for patients with hepatitis C virus (HCV) final stages of infection; even after successful transplantation, the disease remains a clinical and therapeutic challenge for both the patient and the doctor [5].

Approximately 150 million people worldwide (3% of the world population) suffer from hepatitis C [6], and unmanaged viral infection is one of the leading causes of liver cirrhosis and hepatocellular carcinoma (HCC) [7]. However, considering the natural tendency towards malignancy or hepatitis C Cirrhosis, the onset of liver decompensation rapidly orients the disease management toward liver transplant [8].

Treatment with direct-acting antivirals (DAAs) significantly improved the treatment of chronic hepatitis C, and the association of interferon-free agents has proved to be a better tolerated and more efficient solution [9] for post-liver transplant patients as well as for patients on the waiting list for liver transplant [10]. Not all current regimes are suitable for patients awaiting liver transplant because one of the objectives for this particular category is improving, to some extent, the liver function [11] (and, as cynical as it sounds, may result in de-listing), but also lowering the viremia to prevent the graft infection [12,13,14,15].

In an extensive review, Lens et al. underlined the clear indication of interferon-free treatment before liver transplant, with inerrant complications due to the treatment’s uncertain duration. The authors question the clarity of the post-transplant interferon-free treatment guidelines, as the drug interactions between antiviral therapy and immunosuppressants can put the patient at risk but are still the “weapon of choice” for both transplanted or waiting-listed patients with hepatitis C [16]. There are, however, controversial data regarding the risk of developing hepatocellular carcinoma (HCC) in patients receiving DAAs as a singular treatment, without a liver transplant, for hepatitis C [17,18].

Several scores are currently available to predict outcomes following liver transplantation, including the model for end-stage liver disease (MELD), survival outcome following liver transplantation (SOFT), and balance of risk (BAR) scores [19]. However, an ideal and universally accepted score to predict adverse events in liver transplant recipients with hepatitis C is lacking.

Machine learning (ML)-based solutions are novel and promising tools that could lead us closer to an ideal score. These methods have been used to predict complications in various types of transplantation, including heart, lung, liver, and kidney transplantation. The results have been promising; ML models were trained to predict acute rejection, infection, and other complications in heart and kidney transplant recipients. In lung transplantation, they proved to be reliable in predicting chronic lung allograft dysfunction, which is a common complication in this clinical setting. For liver transplant recipients, artificial intelligence algorithms predicted postoperative complications such as hepatic artery thrombosis and primary non-function, which are common and serious complications in this category of patients. Overall, the results of ML methods to predict complications in transplantation show promise and have the potential to improve patient outcomes. However, no study focused on the vulnerable and risk prone category of hepatitis C populations receiving liver transplant.

We aimed to establish and validate the first-ever ML model to predict short-term outcomes of hepatitis C patients who underwent liver transplantation. Our approach is meant to demonstrate that artificial intelligence and ML methods can significantly contribute to the advancement of clinical management in hepatitis C-liver transplant recipients.

## 2. Materials and Methods

### 2.1. Study Population

Our study was conducted on 90 hepatitis C patients who underwent liver transplantation. Surgical interventions were performed within the Surgery Department of the Sf. Spiridon Hospital Iași and Fundeni Clinical Institute, Bucharest, Romania, between September 2000 and April 2017. All included patients were eligible candidates for liver transplantation according to the national criteria: a MELD-sodium (MELD-Na) score > 15 or a diagnosis of HCC. Patients with an uncertain diagnosis of HCV infection in their history were excluded.

All patients provided written informed consent. The study has full ethical approval from Gr. T. Popa University of Medicine and Pharmacy Iași (158/27.02.2022), Sf. Spiridon Hospital and Fundeni Clinical Institute Ethics Committees. No sex-based or racial/ethnic-based differences were present.

### 2.2. Study Design: Definitions, Transplantation Techniques, and Follow-Up

We conducted a retrospective observational two-center cohort study.

All patients were hospitalized to perform liver transplantation after a rigorous selection of the cases. Patients had to meet the national criteria for transplantation, as stated above. The MELD-Na score predicted early mortality in cirrhosis patients and was calculated using online tools (https://www.mdcalc.com/calc/1754/meldna-meld-na-score-liver-cirrhosis)(accessed on 1 November 2022). The diagnosis of HCC was established by imaging (computer tomography/magnetic resonance imaging) or histopathological findings, according to the American Association for the Study of Liver Diseases [20].

Three types of liver transplantation techniques were performed for this study: whole deceased transplantation, deceased segmental transplantation, and living donor segmental transplantation.

All patients received lifelong follow-up, as recommended by the European Association for the Study of the Liver (EASL) [21].

### 2.3. Data Collection

The study database contains the predicted outcome’s value and a series of variables selected as predictors for each patient.

The outcome to be predicted by our model is the presence or absence of postoperative complications in the first month after surgical intervention. Short-term postoperative complications were defined to be: sepsis; variceal hemorrhage; renal dysfunction; respiratory failure; disseminated intravascular coagulation; septic shock; multiple organ dysfunction syndromes; cardiac arrest; multiple systems organ failures; post-transplant lymphoproliferative disorder; biliary anastomosis stenosis—endoscopic stent; tumor recurrence, peritoneal carcinomatosis; HCV reinfection; graft infection with the hepatitis B virus; idiopathic transverse colon necrosis; bone and brain metastases; necrotizing pancreatitis; hepatic artery thrombosis; hemoperitoneum; primary non-functioning of the transplant graft; or common bile duct necrosis.

The following 14 clinical and laboratory pre-transplant parameters were collected and used as predictors: age, sex, blood type (ABO, RH), the diagnosis which prompted the need for liver transplantation (1—hepatitis C cirrhosis; 2—hepatitis C cirrhosis and HCC; 3—coinfection of HCV, hepatitis B virus and hepatitis D virus; 4—HCC associated with the coinfection of HCV, hepatitis B virus and hepatitis D virus), age at diagnosis, MELD-Na score, alpha-fetoprotein, pre-transplant antiviral treatment, liver re-transplantation, total bilirubin, platelet count, albumin, international normalized ratio, and the presence of ascites.

### 2.4. Machine Learning Approach: Model Training

The dataset was used to train a deep-learning model for predicting short-term postoperative complications based on the clinical and laboratory parameters defined above. The model was built and validated using the following Python libraries: Tensorflow 2.8.0, Numpy 1.22.3, Pandas 1.4.2, and Matplotlib 3.3.2.

The database contained no missing values.

Firstly, numeric predictors from the dataset were normalized according to the formula:


$$X_{\mathrm{normalized}}=\frac{X-X_{\min}}{X_{\max}-X_{\min}}$$

Further, we randomly divided the dataset into a training set (80 records) and a validation set (10 records).

The training set was used to build the deep learning classifier based on a sequential model defined by three dense layers of sizes 64, 32, and 8 neurons, with a dropout of 0—0.2. The sigmoid function was used as the activation function for the last layer, while the other layers were activated by the rectified linear unit (ReLU) function:


$$\mathrm{ReLU}\sim f(x)=\{\begin{array}{ll} x & x>0\\ 0 & x<=0 \end{array}$$

Adam [22], an optimization algorithm specific to deep learning models, was used instead of the classical stochastic gradient descent procedure to update network weights in the training data iteratively.

The learning rate was set to be 10^−3^, and the number of epochs was 85. We identified the correct number of epochs following an iterative process. We started by setting the number of epochs to 45 (3 times the number of columns in our data). We incremented the number of epochs as long as the model was still improving (lower loss, higher accuracy).

Mean absolute error (MAE), loss and accuracy are the metrics used to evaluate the model’s performance on the training set based on the output probabilities.

The validation set was finally interrogated to evaluate the model’s performance on independent data that did not participate in the training process. Accuracy, the area under the receiver operating characteristic curve (AUC) and F2 are the metrics used to assess the performance of the validation set.

## 3. Results

### 3.1. Patients’ Characteristics

Of all 90 patient records, 51 (56.7%) were male and 39 (43.3%) were female. The age range of the participants was 12–68. The training and validation sets’ clinical characteristics and laboratory findings are summarized in Table 1 and Table 2, respectively.

### 3.2. Training and Validation of the ML Model

**Figure 1:**
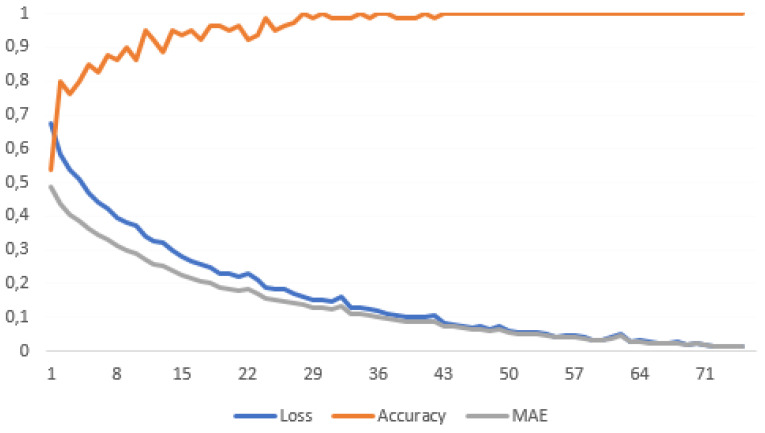
The plot of loss, accuracy and MAE versus the number of epochs during the training step.

We first trained the model on the training set. To ensure that no overfitting was happening and that the model performed well on the training data, we monitored its performance as illustrated in Figure 1. The figure proves that, once the number of epochs increases, the errors’ values (loss and MAE) decrease and accuracy increases, as expected.

Throughout the 85 training epochs, the model achieved a very good performance with the accuracy ranging between 99.76% and 100%. After achieving an excellent performance of the model during the training step, we moved to testing the model on an independent patient cohort.

After testing the model on the validation set, the deep-learning classifier confirmed the performance in predicting postoperative complications, achieving an accuracy of 100% on unseen data. The model also obtained an AUC and an F2 score of one on the validation set.

## 4. Discussion

To the best of our knowledge, the present study is the first to establish and validate an ML model for the short-term outcomes prediction of hepatitis C patients who underwent liver transplantation.

The developed ML model had an excellent accuracy for postoperative outcomes prediction in the training cohort (99.76–100%) and in the validation cohort (100%). Therefore, reported results could be a solution for better identifying high-risk hepatitis C patients in the pre-liver transplant setting. Moreover, the proposed model does not constitute a preclusion instrument for liver transplantation, but rather a reliable tool to guide a more intensive follow-up protocol in high-risk patients. As well as the ML model, we provided data regarding postoperative complications in this particular subset of patients.

Available data on artificial intelligence usefulness in liver transplant patients with hepatitis C are limited. Concerning this issue, an early study investigated the opportunities provided by artificial neural networks for significant liver fibrosis prediction [23]. In 123 hepatitis C-infected liver transplant patients (training cohort), the proposed algorithm had an excellent predictive value for liver fibrosis (AUC 0.87, 95% CI, 0.84–0.90). In addition, the performance was even better in the validation cohort (AUC 0.93, 95% CI, 0.86–0.97) [23].

Anticipated success in treating HCV raises the hope of reducing the rate of complications, preventing progression to liver decompensation and progression to hepatocarcinoma. On the other hand, efforts have been made to improve liver function in patients with decompensated cirrhosis, and liver transplantation remains the only opportunity in end-stage disease [24]. Thus, patients awaiting liver transplantation are frequently frail, and a better stratification of the postoperative risk of adverse outcomes is required.

The importance of the artificial intelligence approach for outcomes prediction following liver transplantation was highlighted in a Korean registry involving 785 deceased donor transplant recipients [25]. Among all analyzed methods, the random forest model had the best predictive power for survival at one month, 3, and 12 months (respectively, AUC = 0.80, AUC = 0.85, and AUC = 0.81). Traditional risk scores such as MELD and BAR performed significantly lower in survival prediction (at one month, AUC = 0.64 and AUC = 0.68, respectively). Therefore, ML methods might be a better solution than classic models for outcomes prediction in patients with liver transplantation. Nevertheless, only 6.4% of patients (*n* = 50) had hepatitis C, and subgroup analysis in this particular subset of patients was not performed [25].

Patients undergoing LT for virus C related cirrhosis have one of the highest short- and long-term mortality compared to other etiologies [21]. Studies have also shown that patients with hepatitis C-related cirrhosis have higher rates of postoperative complications, such as primary non-function, hepatic artery thrombosis, and recurrent hepatitis C infection, compared to patients with other etiologies [21]. These complications can contribute to higher short- and long-term mortality rates in patients with hepatitis C-related cirrhosis who undergo liver transplantation. However, none of the AI-based studies published so far focused on predicting complications amongst hepatitis C patients. Moreover, their datasets contain a very low number of patients with HCV infection. This makes their predictions on this specific population unreliable.

Further research on the ML approach in hepatitis C patients is warranted, as 85% of infected patients develop chronic hepatitis and 10–20% progress to cirrhosis [26]. Additionally, 7% of patients with cirrhosis will develop hepatocarcinoma [27]. Notably, end-stage hepatitis C is the leading indication for liver transplantation. Patients diagnosed with chronic hepatitis C virus display increased morbidity, the hospitalization rate is high [28], and the mortality rate is three times higher than in the general population [29]. Although hepatitis C virus transmission has been significantly reduced and prevention strategies are effective [30], patients still represent an economic burden [31].

In the early period after liver transplantation, patients are exposed to a high rate of complications which constitutes a key motivation for our study. The risk of infections represents a possible complication associated with a series of pre-existing comorbidities, age, or obesity. In addition, immunosuppressive treatment administered systemically to transplant recipients could increase the susceptibility of de novo infections or the reactivation of pre-existing latent infections [32]. Usually, infections occurring during the first-month post-liver transplantation are healthcare-associated, donor-derived, or a consequence of organ dysfunction [33].

A recent review of the Organ Procurement and Transplantation Network (OPTN) data from 64,977 patients who underwent liver transplantation identified a 5% and a 10% mortality incidence at 90-day and 1-year follow-up, respectively. Particularly, death associated with cardiovascular/cerebrovascular/pulmonary/hemorrhage was the most common cause of death within the first 21 days (at seven days, 53%) [34]. Thus, identifying these patients with an increased risk of adverse events in early postoperative settings was the main objective of our endeavor.

Our study was conducted on 90 hepatitis C patients who underwent liver transplantation. They all were eligible candidates for the procedure. We conducted a retrospective observational two-center cohort study on patients meeting the national criteria for liver transplantation. Although our deep learning algorithm performed excellently in predicting the most common post-transplantation complications, it should be tested and validated in large cohorts of liver transplant patients before being implemented extensively. In this way, patients’ evolution could be predicted accurately and non-invasively according to a series of pre-transplant variables. Consequently, we could aim to increase the survival rate and the quality of life of liver transplant patients infected with hepatitis C by adopting an appropriate monitoring strategy in the early post-transplant period.

Following extensive external validation, our system could assist doctors in making early lifesaving decisions in day-to-day clinical practice. For instance, high urgent status patients in need of an acute re-transplantation due to hepatic artery thrombosis after LT or primary non-functioning of the transplant graft [21] would greatly benefit from the early prediction of the complications and timely initiation of the re-transplantion procedures. Our software predictions could also prompt the initiation of early antiviral therapy in patients with HCV recurrence after LT. This has a great clinical impact, as HCV infection after LT is characterized by an accelerated fibrotic progression towards chronic hepatitis and cirrhosis [21]. Early treatment followed by sustained viral response significantly improves outcomes in these patients [21]. In the case of the risk of developing renal dysfunction after LT, a practical utility of the implementation of our algorithm could be to assist the doctor in the timely reduction or withdrawal of the nephrotoxic calcineurin inhibitors (CNI) and switch to alternative CNI-free protocols for immunosuppression (according to the grade I recommendation of the EASL guidelines) [21].

Clinical and laboratory parameters before transplantation can be important risk factors for predicting postoperative complications after liver transplant. The evaluation of these parameters can provide valuable information about the patient’s overall health and the potential challenges that may arise during and after the transplant procedure. Studies have shown that certain demographic factors, such as age, gender, and race, can be associated with an increased risk of complications [35]. Older patients, for example, are more likely to have additional medical conditions and a weakened immune system, which can increase the risk of complications. Similarly, there is some evidence to suggest that female gender may be associated with an increased risk of post-liver transplant morbidity and mortality compared to male recipients [35]. However, the reasons for this difference are not well understood and further research is needed to fully understand the relationship between gender and outcomes after liver transplantation. Therefore, it is important for healthcare providers to consider the individual patient’s risk factors, including gender, when assessing their risk for post-liver transplant complications and developing a comprehensive care plan. Additionally, pre-transplant laboratory parameters, such as ABO blood type, can also play a role in predicting the risk of complications [36]. A study showed that patients with an ABO blood type mismatch between the donor and recipient have an increased risk of acute rejection [36]. Therefore, the pre-transplant parameters can help in predicting complications, allowing for more proactive and tailored management strategies.

The severity and frequency of postoperative complications (especially infections) within 30 days after a liver transplant can be higher compared to long-term complications [33]. While some of these complications can be severe and potentially life-threatening, they are usually more treatable and reversible compared to long-term complications if detected early and managed appropriately. Thus, the vital need for a predictive tool for short-term postoperative complications arises.

Our ML model trained on data from hepatitis C transplant recipients could be adapted for use in predicting complications in hepatitis B transplant recipients by retraining it on relevant data from this other population, or by modifying the model architecture to better handle the differences in underlying biology and disease progression between the two types of hepatitis (for example, HCV replicates more rapidly; the body’s immune response to HCV and hepatitis B virus can differ as some individuals can clear hepatitis B virus on their own, while this is less common with HCV; and chronic hepatitis C can progress more rapidly to cirrhosis and liver cancer). However, this would need to be undertaken carefully and be validated on a separate dataset to ensure its accuracy.

### Limitations

Firstly, the size of our dataset is small and from the same two centers as the independent validation set, as the training set prompts more external validation with data from other centers. Secondly, the retrospective nature of our study predisposes us to selection bias which can be overcome in future prospective studies on larger datasets. Thirdly, our ML system cannot yet detect if the patient will incur low or high prevalence of postoperative complications. However, this ability will be incorporated in the model in our future studies. Fourthly, the results of our study carried out in Romania may not be directly applicable to other regions, and it may be necessary to develop and validate separate models for different regions or demographic groups. The outcomes of the ML model may differ if the study is carried out in different parts of the world, due to differences in demographic factors such as age, gender, race, and socioeconomic status, as well as differences in healthcare systems, medical practices, access to medical technology, lifestyle and environmental factors. In addition, there may also be differences in the prevalence and severity of hepatitis C and its complications in different regions of the world, which can impact the results of the study.

## 5. Conclusions

To the best of our knowledge, this is the first ML-based study to provide an ML algorithm for predicting postoperative complications in liver transplant recipients infected with hepatitis C. We successfully developed an ML model to predict postoperative complications following liver transplantation. The model demonstrated excellent performance and holds promise for future clinical applications and research to accurately predict post-transplant short-term evolution. Consequently, the present study constitutes the foundation for the careful and non-invasive identification of high-risk patients who might benefit from a more intensive post-operative monitoring strategy. Nevertheless, the results should be confirmed in extensive prospective studies to facilitate the implementation of ML risk stratification in patients scheduled for liver transplantation.

## Figures and Tables

**Table 1:** Characterization of the numerical and categorical variables included in the training set (80 patients).

Numerical Variables										
	Mean	Standard Deviation	Median	Median Absolute Deviation				Min		Max
Age	47.93	8.74	48	5.19				12		68
Age at diagnosis	37.89	8.26	40	1.48				2		51
MELD-Na	16.35	6.63	16	5.93				5		37
AFP (ng/mL)	127.03	358.30	10.43	10.27				0.01		2000
Categorical variables										
	Categories definition		Number of occurrences of each category							
			1		2	3	4		5	
Sex	1 (female), 2 (male)		35		45	-	-		-	
AB0 blood type	1 (0), 2(A), 3 (B), 4 (AB)		16		43	12	9		-	
Rh group	1 (−), 2 (+)		10		70	-	-		-	
The diagnosis that prompted LT	1 (hepatitis C cirrhosis), 2 (hepatitis C cirrhosis and HCC), 3 (coinfection of HCV, hepatitis B virus, and hepatitis D virus), 4 (HCC associated with the coinfection of HCV, hepatitis B virus, and hepatitis D virus)		55		23	1	1		-	
Total bilirubin (mg/dL)	1 (0.2–1.20), 2 (1.2–4), 3 (4–8), 4 (>8)		16		39	22	3		-	
Platelet count (×10^3^/μL)	1 (0–20) 2 (20–40) 3 (40–80) 4 (80–150) 5 (150–400)		9		28	27	11		4	
Albumin (g/dL)	1 (≤2.8), 2 (2.8–3.5), 3 (≥3.5)		5		50	25	-		-	
INR	1 (˂1.7), 2 (1.7–2.2), 3 (˃ 2.2)		5		56	19	-		-	
Pre-transplant antiviral treatment	1 (none), 2 (interferon), 3 (interferon free)		1		68	11	-		-	
Liver re-transplantation	1 (No), 2 (Yes)		74		6	-	-		-	
Ascites	1 (No), 2 (Yes)		20		60	-	-		-	
Postoperative complications	1 (No), 2 (Yes)		58		22	-	-		-	

**Table 2:** Characterization of the numerical and categorical variables included in the validation set (10 patients).

Numerical Variables										
	Mean	Standard Deviation	Median		Median Absolute Deviation			Min		Max
Age	47.93	47.1	10.24		47			12.60		32
Age at diagnosis	37.89	36.2	9.81		36.5			7.41		20
MELD-Na	16.35	17.9	6.47		15			5.19		11
AFP (ng/mL)	127.03	53	69.61		2			0.01		0.1
Categorical variables										
	Categories definition		Number of occurrences of each category							
			1	2		3	4		5	
Sex	1 (female), 2 (male)		4	6		-	-		-	
AB0 blood type	1 (0), 2(A), 3 (B), 4 (AB)		3	2		3	2		-	
Rh group	1 (−), 2 (+)		4	6		-	-		-	
The diagnosis that prompted LT	1 (hepatitis C cirrhosis), 2 (hepatitis C cirrhosis and HCC), 3 (coinfection of HCV, hepatitis B virus, and hepatitis D virus), 4 (HCC associated with the coinfection of HCV, hepatitis B virus, and hepatitis D virus)		6	4		0	0		-	
Total bilirubin (mg/dL)	1 (0.2–1.20), 2 (1.2–4), 3 (4–8), 4 (>8)		2	2		3	3		-	
Platelet count (×10^3^/μL)	1 (0–20) 2 (20–40) 3 (40–80) 4 (80–150) 5 (150–400)		2	3		3	2		-	
Albumin (g/dL)	1 (≤2.8), 2 (2.8–3.5), 3 (≥3.5)		3	5		2	-		-	
INR	1 (˂1.7), 2 (1.7–2.2), 3 (˃ 2.2)		3	5		2	-		-	
Pre-transplant antiviral treatment	1 (none), 2 (interferon), 3 (interferon free)		1	4		5	-		-	
Liver re-transplantation	1 (No), 2 (Yes)		8	2		-	-		-	
Ascites	1 (No), 2 (Yes)		5	5		-	-		-	
Postoperative complications	1 (No), 2 (Yes)		6	4		-	-		-	

## Data Availability

The Python source code implementing our model will be available from the corresponding author upon request.
